# Plate-Focusing Based on a Meta-Molecule of Dendritic Structure in the Visible Frequency

**DOI:** 10.3390/molecules23061323

**Published:** 2018-05-31

**Authors:** Suna Cheng, Di An, Huan Chen, Xiaopeng Zhao

**Affiliations:** Smart Materials Laboratory, Department of Applied Physics, Northwestern Polytechnical University, Xi’an 710129, China; 15991701865@163.com (S.C.); di__an@163.com (D.A.); chenhuan151@mail.nwpu.edu.cn (H.C.)

**Keywords:** dendritic structure, anomalous reflection, anomalous refraction, visible light, plate-focusing metasurface

## Abstract

To study the potential application of metasurfaces in lens technology, we propose a dendritic meta-molecule surface (also referred to as a dendritic metasurface) and realize the focusing effect in the visible spectrum through simulations and experiments. Using asymmetric dendritic structures, this metasurface can achieve distinct broadband anomalous reflection and refraction. When the metasurface is rotated by 180° around the *z* axis, anomalous reflection and refraction in vertically incident optical waves are in opposite directions. Considering this feature, a metasurface is designed to achieve a prominent plate-focusing effect. Samples with a transmission peak of green light at 555 nm, yellow light at 580 nm, and red light at 650 nm were prepared using bottom-up electrochemical deposition, and the focus intensity of approximately 10% and focal length of almost 600 µm were experimentally demonstrated.

## 1. Introduction

The electromagnetic properties of metamaterials, which are specific media consisting of artificial composite materials, can be designed according to preference [1,2,3]. Metamaterials display abnormal physical properties, such as negative refraction [4,5], negative permittivity, and negative permeability [6,7]. These materials present potential applications in super lens imaging, high-sensitivity sensing, and stealth technology. However, the development of metamaterials in near-infrared and visible-light spectra was significantly limited by preparation technology, high loss, and other factors until the emergence of metasurfaces. As 2D metamaterials, metasurfaces consist of a quasi-period array with subwavelength thickness [8,9]. When a beam is irradiated on a metasurface, the elements undergo different phase changes through resonance to control the electromagnetic wavefront. A metasurface can also freely regulate the amplitude, phase, polarization mode, and beam shape of reflected or transmitted waves. The use of metasurfaces exhibits significant advantages over the traditional method of controlling electromagnetic waves by phase accumulation [10,11,12,13,14,15]. Metasurfaces present numerous optical functions, such as anomalous reflection, anomalous refraction, dispersive imaging, quarter-wave plates, and optical focusing.

The use of metasurfaces has received substantial attention since its emergence. In 2011, Yu et al. [8] presented a metasurface with V-shaped elements to achieve anomalous refraction and applied the generalized Snell’s law for theoretical verification. Researchers have achieved a quarter-wave plate [16], flat lens, and flat vortex plate through different spatial arrangements of unit structures. Metasurfaces substantially simplify the design and size of lenses, thereby allowing beam focus on subwavelength thickness. Pors et al. [17,18] proposed a metasurface consisting of gold nanobricks to achieve continuous phase modulation in the near-infrared band. This metasurface obtains the focusing effect by assembling gold nanobricks. Aieta et al. [19,20] developed a flat metasurface lens to eliminate dispersion by phase compensation. Li et al. [21] proposed achromatic flat optical components via compensation between structure and material dispersions. An achromatic deflector and lens were designed based on silver slits with variable widths within a broadband range of 1000–2000 nm. However, almost all of these metasurfaces were fabricated through top-down lithography or etching approaches, which are unsuitable for practical applications because of their high cost and small sample volume. This difficulty in sample preparation hinders research progress. To address the bottleneck posed by preparing metasurfaces in infrared and visible light, we developed a dendritic meta-molecule surface (that is, a dendritic metasurface) via bottom-up electrochemical deposition fabrication [22,23,24], which is a facile and cheap method. We demonstrated that left-handed materials (LHMs) with unified dendritic unit cells arrayed in a disordered state exhibit negative refraction [25]. On the basis of gold dendritic metasurface, which achieved the focusing phenomenon in the mid-infrared frequency band [26], we designed a silver dendritic metasurface by adjusting the size of the structural units to achieve a plate-focusing effect in the visible light band by simulations and experiments. Numerical simulations confirmed the anomalous reflection and refraction effects over a wide waveband range. Units of branches were assembled to form a silver dendritic metasurface and realize the focusing effect. The experiments demonstrated that this structure can achieve the flat focusing effect in the visible spectrum.

## 2. Anomalous Reflection and Refraction of a Metasurface with Asymmetric Dendritic Structure

### 2.1. Model Structure and Simulation Environment

**Figure 1:**
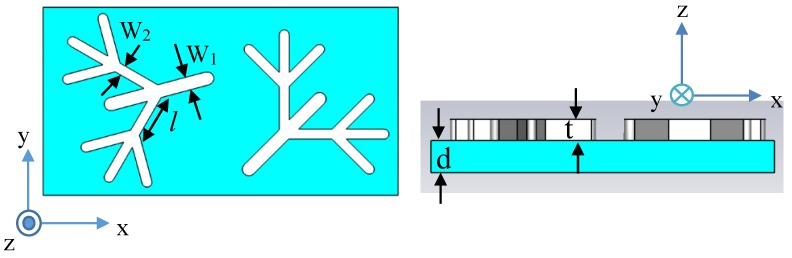
Schematic of the dendritic structure metasurface. The length of the branches is *l* = 90 nm, the width of the main branches is W_1_ = 24 nm and the width of the side branches is W_2_ = 18 nm. The substrate thickness is d = 20 nm and the thickness of the gold branches is t = 40 nm.

A metasurface composed of silver dendritic structures with two randomly arranged branches is shown in Figure 1. The simulated structure with a simple six-unit cell is an example for the actual samples. Other shapes and size combinations for the model unit cell lead to markedly different optical properties, or similar results can be obtained for a range of model structures, such as a metasurface composed of nine kinds of dendritic structures to achieve abnormal Goos-Hänchen (GH) shifts and rainbow trapping effect [24], a metasurface composed of eleven kinds of dendritic structures to perform differential operation [27], and so on. The region illuminated by the laser in the experiment is much larger than the size of the model cell, which sets an additional limitation to the possibility of modeling the behavior of real samples.

To determine the shapes of the optical wavefronts formed by the dendritic structure metasurfaces, we performed numerical simulations using the commercial software Finite Different Time Domain (FDTD) on the basis of the finite difference time domain method. In our simulations, the optical constants of silver were set at those of the Drude model. Silver dendritic structures were assumed to be designed on a silica substrate (*n* = 1.46) and illuminated by a normally incident plane wave of *y* polarization. The boundary conditions along the *x* and *y* axes were set as infinite periodic boundaries, and the boundaries in the *z* axis direction were surrounded by perfectly matched layers (PML). The optimized dimensions of the dendritic structure were as follows: *l* = 90 nm, d = 20 nm, t = 40 nm, W_1_ = 24 nm, W_2_ = 18 nm, *l*_a_ = 640 nm, and *l*_b_ = 340 nm. *l* denotes the length of the branches shown in Figure 1, d denotes the substrate thickness, t denotes the thickness of the gold branches, W_1_ is the width of the main branches, W_2_ is the width of the side branches, and *l*_a_ and *l*_b_ are the length and width of the substrate, respectively. Compared with existing analysis modeling approaches [28,29,30], the results obtained using our calculation method can better reflect the properties of the metasurface, and the effective parameters are not the intrinsic properties of the metasurface [31]. The model proposed here can be prepared by using a simple and low-cost electrochemical deposition method.

### 2.2. Anomalous Reflection and Refraction of Dendritic Structure Metasurface

**Figure 2:**
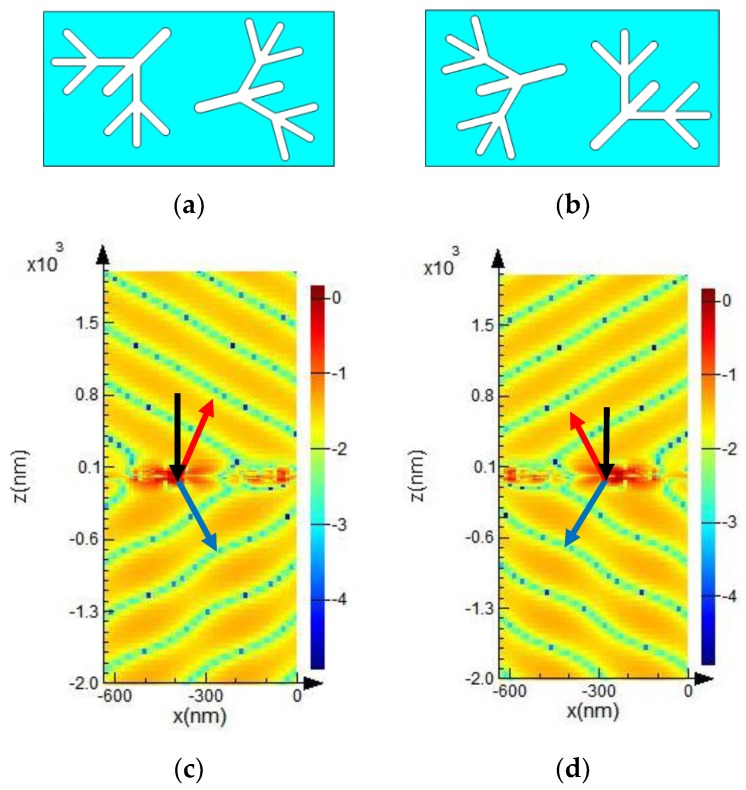
Schematic of one group of structural units (**a**,**b**) and the simulated results (**c**,**d**). (**b**) is obtained by rotating (**a**) 180°around the *z*-axis; (**c**,**d**) scattered electric field distribution *E_x_* of (**a**,**b**), respectively.

We designed an asymmetric structure metasurface (Figure 2a) and simulated the optical responses, namely, reflection and refraction, of the metasurface with a dendritic structure. The metasurface shown in Figure 2b was obtained by rotating the structure in Figure 2a by 180° around the *z* axis. The results showed that the dendritic structure metasurface achieved anomalous reflection and refraction within the broadband range of 521.7–638.3 nm. Only the results at 547.9 nm were presented in this paper as an example. However, the deflection angles vary at different frequencies. Given the wide frequency range, the angles at other frequencies cannot be listed in this paper individually. Figure 2c,d shows the results of the *E_x_* component of the scattered electric fields for the dendritic structure metasurfaces shown in Figure 2a,b at 547.9 nm, respectively. The metasurface was located on the *xoy* plane of *z* = 0. Black, blue, and red arrows indicate the directions of incidence, refraction, and reflection, respectively. The anomalous responses (reflection and refraction directions) shown in Figure 2c,d were opposing.

**Figure 3:**
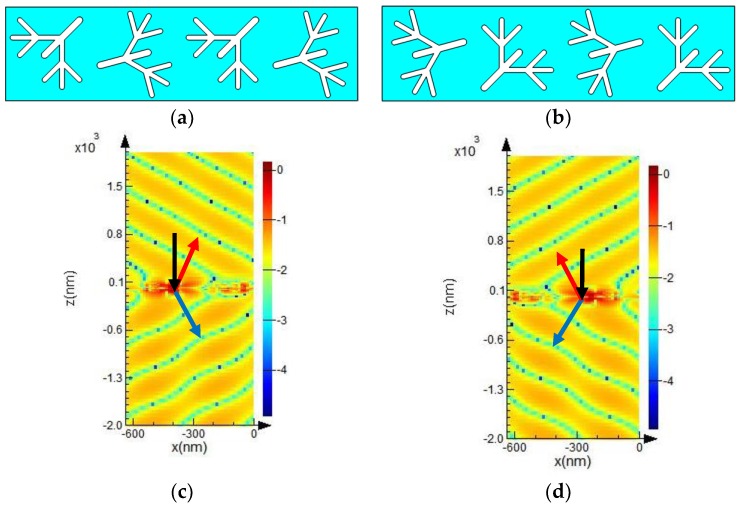
Schematic of the two groups of structural units (**a**,**b**) and the simulated results (**c**,**d**). (**b**) is obtained by rotating (**a**) 180° around the *z*-axis; (**c**,**d**) scattered electric field distribution *E_x_* of (**a**,**b**), respectively.

Related experiments and theories [32,33,34] show that metamaterial is a weak interaction system, that is, the interaction between each dendritic meta-molecule unit in the designed metasurface is weak, referring to the weak near-field interaction. Subsequently, we arranged two groups of structural units side by side. The model diagrams are shown in Figure 3a. The metasurface shown in Figure 3a is a combination of the metasurfaces shown in Figure 2a,b. This metasurface also exhibited anomalous reflection and refraction within the broadband range of 521.7–638.3 nm. Only the result at 547.9 nm is shown in Figure 3c for the same reason as stated above. Figure 3b was obtained by rotating the structure in Figure 3a by 180° around the *z* axis. Figure 3c presents the anomalous refraction and reflection results of the dendritic metasurface of Figure 3a. For comparison, the corresponding result of Figure 3b is plotted in Figure 3d. Comparing Figure 2c and Figure 3c, it can be seen that both the models shown in Figure 2a and Figure 3a can generate anomalies, but the model shown in Figure 3a can produce larger angles of abnormal refraction and reflection. Similarly, the model shown in Figure 3b can produce larger angles of abnormal refraction and reflection compared with those produced by the model shown in Figure 2b. These results prove that anomaly still exists with increasing number of structural units and that the scattered fields are different but similar.

### 2.3. Physical Mechanism of Anomalous Effect

Theories and experiments have proven that a dendritic meta-molecule is actually a combination of a metal rod and split-ring resonator that can simultaneously achieve negative *ε* and negative *μ* in microwave and infrared [23,25], respectively. The paper [26] has demonstrated that a dendritic metasurface responding in infrared can be designed on the basis of dendritic metamaterial. Additionally, the simulated results of the paper indicate that the transmission of light conforms to the generalized Snell’s law and the metasurface can produce focusing phenomena in infrared. Here, we extend the relevant results to visible light.

## 3. Anomalous Reflection and Refraction in the Dendritic Model to Achieve Focusing Effect

**Figure 4:**
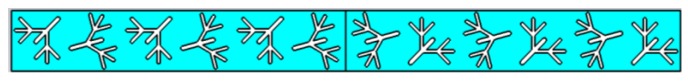
Metasurface model composed of six groups of units.

The abnormal phenomena of the dendritic structure metasurface were in opposite directions before and after rotation. We selected optimized branch units with sizes similar to the samples but with different orientations to form the building blocks. The building blocks were combined to form a dendritic metasurface that is close to the distribution of dendritic structures in the actual sample to achieve focusing effect. First, six unit groups were used as a model (Figure 4). The right half structure was obtained by rotating the left half structure by 180° around the *z* axis. We simulated the optical response of this structure at the visible frequency and obtained the scattered electric field mode of ${\left|E_{x}\right|}^{2}$.

The number of dendritic meta-molecule units in the sample was very large (the sample with an area of 1 cm^2^ contained approximately 10^9^ dendritic units). The dendritic units used in the simulation were only equivalent to a portion of units in the sample. The distribution of these structures appeared disorderly but statistically quasi-periodic and symmetric along the *x* and *y* directions. Therefore, the sample can be regarded as consisting of numerous ordered structural elements similar to those in the simulation. The simulation results (focusing effect on the *x*–*z* plane) of the metasurface composed of many pairs of dendritic structures presented the real situation of the sample, which was also proven experimentally. Thus, the *y*–*z* profile is expected to be the same as that in the *x*–*z* plane. The prepared sample is a quasi-periodic dendritic metasurface rather than a completely disordered structure; in simulation we continuously increase the number of units and combine them in order to make the designed metasurface deviate from the ideal periodic structure. Ultimately, a quasi-periodic dendritic metasurface model is obtained. This model is an example to prove that the quasi-periodic dendritic metasurface can achieve anomalous optical response. Therefore, the disordered dendritic structures do not need to be set.

**Figure 5:**
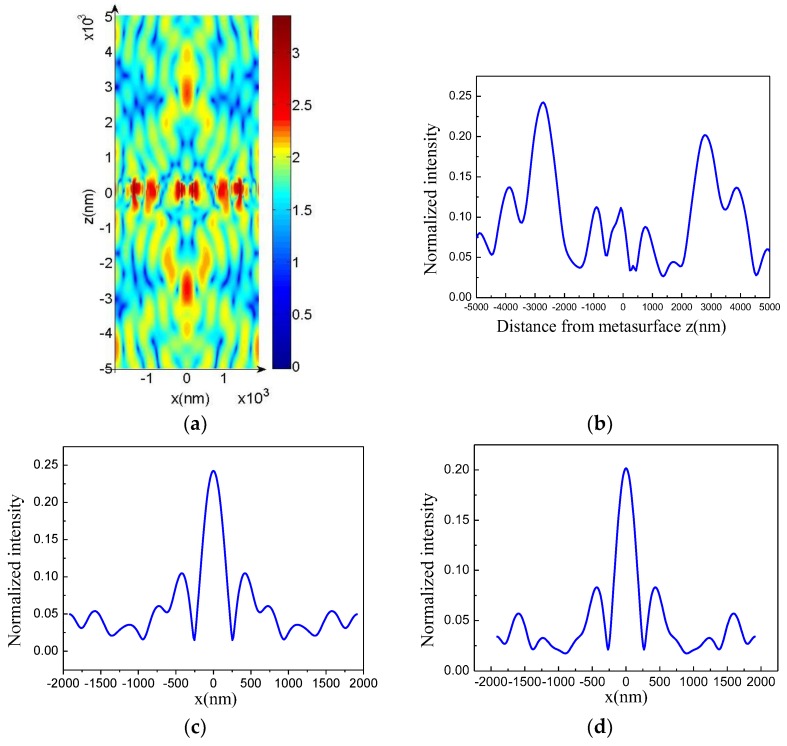
(**a**) Distribution of ${\left|E_{x}\right|}^{2}$ at 564.2 nm; (**b**) intensity as a function of *z*; (**c**,**d**) intensity as function of *x* at *z* = −2608 nm and *z* = 2659 nm, respectively.

According to simulation results, when the normal incident plane wave illuminated the structure, the focusing effect occurred in the reflection (positive direction of the *z* axis) and transmission directions (negative direction of the *z* axis) in the wavelength range of 560.7–582.5 nm. Figure 5a shows the results of the ${\left|E_{x}\right|}^{2}$ component of the dendritic metasurface shown in Figure 4 at 564.2 nm. The normalized intensity at different distances from the metasurface (i.e., *z* axis) when *x* = 0 is shown in Figure 5b. Each side of the metasurface showed an intensity peak. The intensity in the negative half axis was slightly higher than that in the positive half axis. Specifically, the focusing intensity of transmission was slightly higher than that of reflection. Then, we changed the *z* value to examine the relationship between intensity and *x*. Light intensity reached the peak value at *z* = −2608 nm and *z* = 2659 nm (Figure 5c,d, respectively). As shown in Figure 5a–d, the focal lengths of the transmission and reflection were 2608 and 2659 nm, respectively.

**Figure 6:**
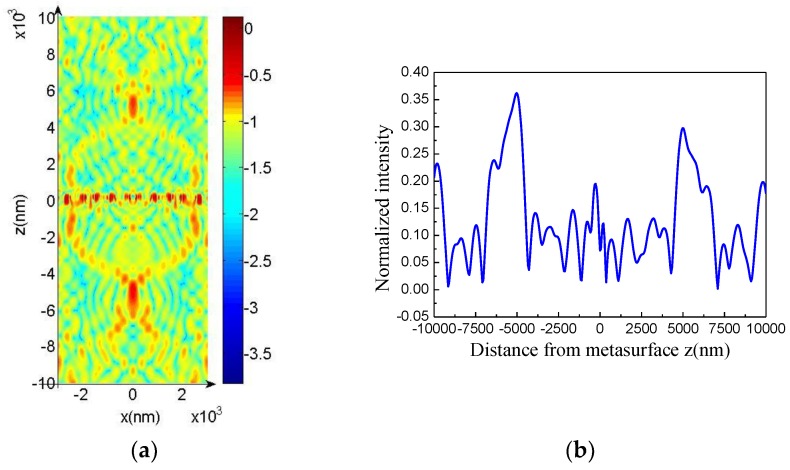

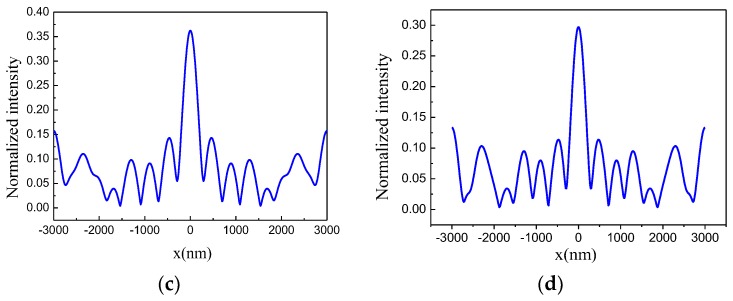
(**a**) Distribution of ${\left|E_{x}\right|}^{2}$ at 568.2 nm; (**b**) intensity as a function of *z*; (**c**,**d**) intensity as a function of *x* at *z* = −4930 nm and *z* = 4892 nm, respectively.

The focusing capability of a metasurface depends on the numerical aperture, which is expressed as NA = sin [tan^−1^ (*D*/2*f*)], where *D* is the width of the metasurface. Thus, we increased the group number of units to increase the width of the metasurface and ultimately increased NA. We selected the metasurface composed of 10 groups of units. The five groups on the right half were obtained by rotating the five groups on the left half by 180° around the *z* axis. When the normal incident plane wave illuminated the structure, the reflected and transmitted light individually crossed, overlapped, and developed into a bright spot in the range of 555.6–588.2 nm. Focusing results at 568.2 nm are shown in Figure 6. Figure 6c,d indicate that the light intensity reached peak value at *z* = −4930 and 4892 nm, respectively. This result indicates that the focal lengths of the transmitted and reflected light were 4930 and 4892 nm, respectively. The results in Figure 6 are similar to those in Figure 5. However, focal intensity is enhanced, focal wavelength range is widened, and focal length is increased when the width of the metasurface increases. Due to the strong electromagnetic resonance [35] in the dendritic structures, in Figure 5a and Figure 6a it can be seen that at *z* = 0 (that is, at the location of the dendritic metasurface) the intensities of electric fields are very large and the electric fields are localized in the dendritic metasurfaces [36], and then the phase and amplitude of the light wave passing through the metasurface are changed.

## 4. Sample Preparation and Testing

The sizes of LHMs are much smaller than their working wavelength. Therefore, sample preparation in the light spectrum is challenging. Traditional top-down lithography or etching approaches are unsuitable for practical applications because of their high cost and small sample volume. The key point is to identify a convenient and low-cost method to prepare LHMs. A silver dendritic structure is a fractal structure formed in a non-equilibrium state. The structure exhibits numerous unusual properties. When the sizes of the prepared dendritic structures are in nanometer scale, the metasurface composed of these dendritic structures will produce various abnormal optical responses, such as light trapping [24], differential operation [27], plate focusing, etc. We used this method of bottom-up electrochemical deposition to prepare silver dendritic structures of nanoscale morphology, and the plate-focusing effect in the infrared band was achieved [23].

**Figure 7:**
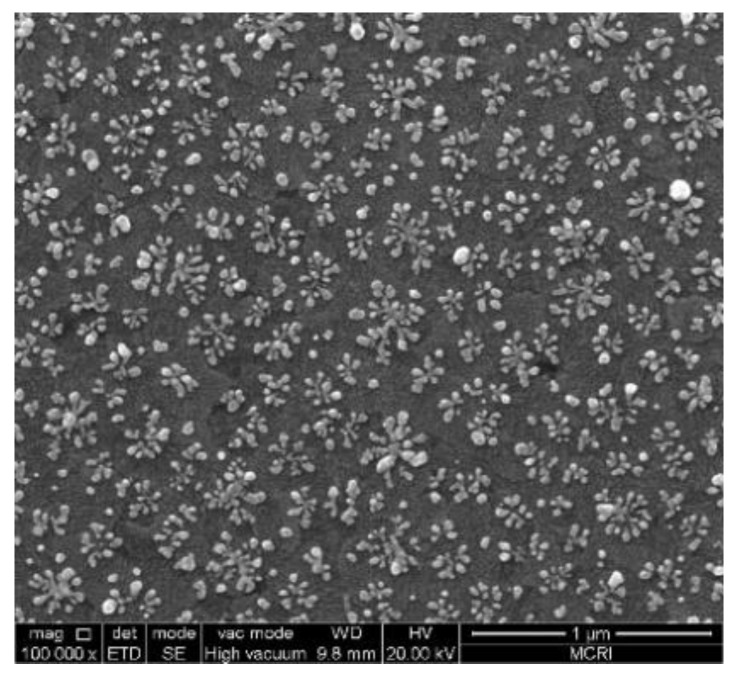
High-resolution SEM image of silver dendritic structures.

In the experiment, electrochemical deposition was used to grow single-layer nanoscale silver meta-molesules of dendritic structure [37] on conductive glass. Indium Tin Oxide (ITO) conductive glass with a specification of 50 mm × 13 mm × 1 mm was used as the cathode, and a smooth silver plate was used as the anode. The two electrodes were separated by two parallel PVC insulating boards with thickness and spacing of 0.625 and 10 mm, respectively. The electrolytes used in the three samples were mixed with a 0.1 mg/mL silver nitrate solution and a 0.12 g/mL polyethylene glycol (PEG-20000) solution. The total experimental process was carried out in an ice bath. By controlling the temperature of the deposition zone at 0 °C to 15 °C, a silver dendritic metasurface with good growth condition and visible light-band response can be obtained. The control of the temperature is mainly related to the distance between the deposition area and ice and the placement time. On the basis of these data, we adjusted the constant voltage applied between the ITO conductive layer and the silver plate and duration of the constant voltage (that is, the duration of the deposition process) to (0.9 V, 60 s), (0.9 V, 45 s), and (0.9 V, 90 s) respectively in the experiment, and samples responding in green, yellow, and red bands can be prepared, respectively. We used a homemade ice bath device to control the operating temperature around 2 °C. A scanning electron microscopy image of the silver branches is shown in Figure 7. The figure shows that the prepared silver dendritic metasurface samples possessed similar structures, and only the structural unit size and distribution density of samples with different transmission peaks were different. The sizes of the structural units responding to a certain visible light band are not same, but they are close to the resonant wavelength, as shown in the SEM image. And each branch molecule is formed by numerous nucleation particles uniformly distributed on the ITO substrate. The growth process and the growth environment are almost the same, and self-similarity between structural units finally appears. Thus, the silver dendritic sample made by electrochemical deposition is statistically quasi-periodic. Moreover, the thickness of the silver dendritic structures was approximately 25 nm.

**Figure 8:**
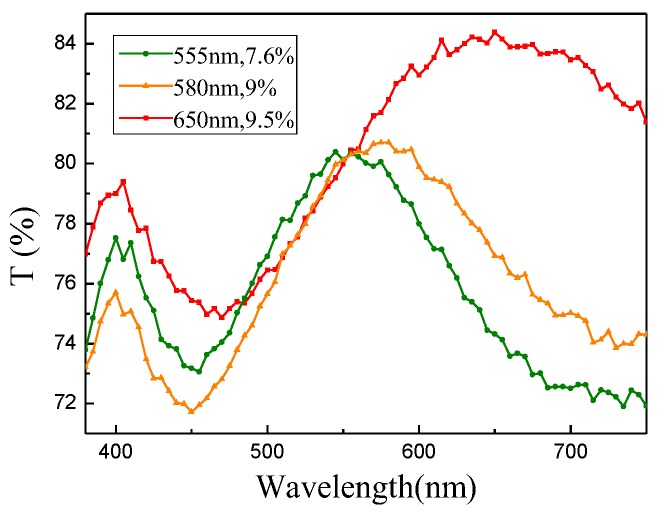
Transmission curves of the samples at three wavelengths and the peak positions of the transmission peaks are 555, 580, and 650 nm, respectively. The percentage in the legend indicates a relative height of transmission peak at the peak wavelength, and the reference is the value of transmission valley at about 450 nm.

**Figure 9:**
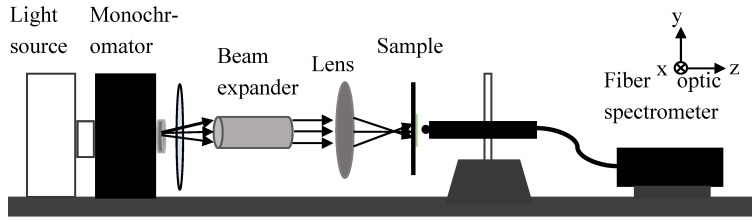
Schematic of the testing device for the focusing behavior.

We selected three samples with transmission peaks at 555, 580, and 650 nm (Figure 8). The focusing experiments were conducted using the apparatus shown in Figure 9. The apparatus was set up on a streak optical platform, and the test was performed in the dark. The light emitted from the source was polychromatic. When it passed through the monochromator, the monochromatic light of the desired wavelength was selected. An aperture was used to adjust the size of the light spot, and a neutral density filter was utilized to adjust light intensity. Light was transformed into a parallel collimation light after passing through the beam expander. Then, the collimated light passed through the lens and converged to a point. The sample was placed at the location closely after the focal point. The position of the optical fiber probe was adjusted to the center of the spot (*x* = 0). The optical fiber probe recorded the intensity of light starting from the surface of the sample and moving from left to right, which is denoted as the *z* direction. At the strongest light intensity position, the optical fiber probe moved to the *x* direction and recorded the light strength. We also measured the intensity of light passing through the glass.

**Figure 10:**
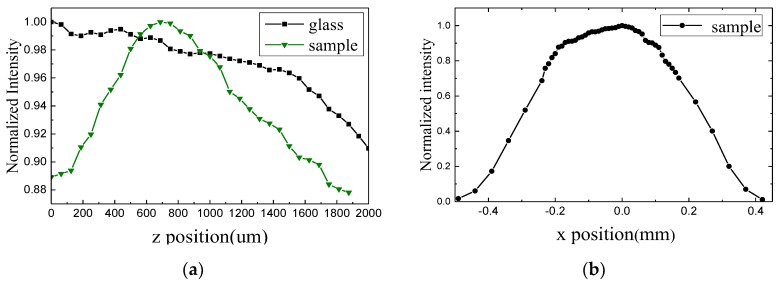
Intensity distribution at 555 nm along the *z* direction (**a**) and *x* direction (**b**).

In the experiment, we selected a given transmission frequency to guarantee that the main structural unit in the sample was resonant at the selected frequency. The sample with a transmission peak at 555 nm was tested after the light wavelength was adjusted to 555 nm by the monochromator. When the samples corresponding to the red and yellow bands were measured, wavelength was adjusted to the resonant peak position of the corresponding samples. We tested the sample with a transmission peak at 555 nm. Figure 10a presents the normalized intensity distribution along the *z* direction. Green and black lines represent the light intensities when light passed through the sample and glass surface, respectively. In the moving process of the optical fiber probe, light intensity passing through the sample initially increased and then decreased, whereas the light intensity through the glass showed a sustained downward trend. Moreover, the thickness of the reference glass was similar to that of the substrate for dendritic structures. Focusing phenomenon occurred only after light passed through the sample. Focusing intensity, which is defined as the difference between the maximum of normalized light intensity in z direction and the normalized light intensity at the starting position (that is, *z* = 0), was 11.3%. And the focal length was approximately 690 µm. Figure 10b shows the intensity distribution along the *x* direction at *z* = 690 µm. 

**Figure 11:**
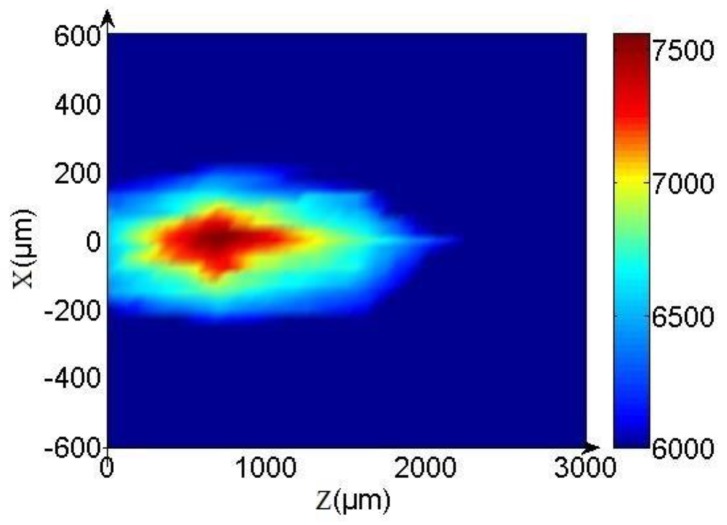
Spatial intensity distribution in *xoz* plane.

To further explain the focusing effect, we measured the intensity on the *xoz* plane at 555 nm. The 2D drawing is shown in Figure 11. In this figure, we can see that the light waves that have passed through the sample clearly converge. The position of maximum light intensity formed by the convergence is at about 690 μm on the *z*-axis, that is, the focal length is about 690 μm, which is consistent with the one-dimensional measurement result.

**Figure 12:**
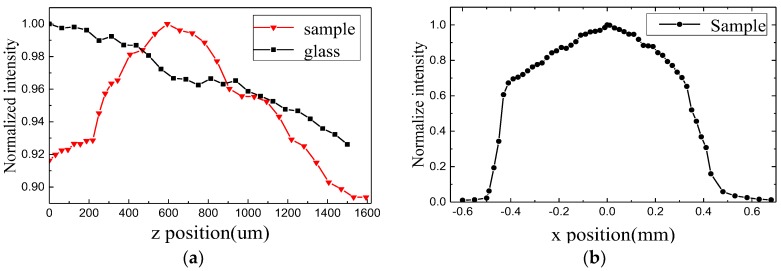
Intensity distribution at 650 nm along the *z* direction (**a**) and *x* direction (**b**).

Then, we tested the sample with a transmission peak of 650 nm. Figure 12a shows the measured light intensity along the line of *x* = 0. The red and black lines represent the sample and glass, respectively. Focusing phenomenon occurred after light was transmitted through the sample. Focus intensity was 8.8%, and focal length was 595 µm. Figure 12b illustrates the intensity distribution along the *x* direction at *z* = 595 µm.

**Figure 13:**
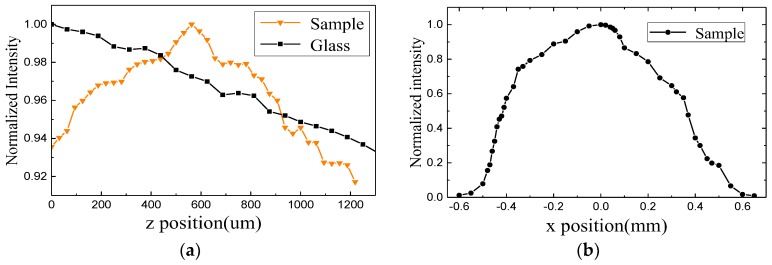
Intensity distribution at 580 nm along the *z* direction (**a**) and *x* direction (**b**).

Next, we tested the sample with a transmission peak at 580 nm. Figure 13a shows the intensity distribution along the *z* direction. The yellow and black lines represent the sample and glass, respectively. The maximum value of light intensity in the *z* direction appeared at 560 µm, and the focus intensity was 6.9%. Figure 13b shows the intensity distribution along the *x* direction at *z* = 560 µm.

Given that the growth condition, including the structure size and distribution density, of the test area of each sample is different, a certain correspondence between the focal length and the transmission peak of the sample may not be present.

## 5. Relationship between the Experiment and Simulation

In the simulation, the metasurface shown in Figure 3a is obtained by combining the two sets of elements shown in Figure 2a,b, then the metasurface shown in Figure 3a is rotated to obtain the metasurface shown in Figure 3b. All these operations are to make the designed metasurface deviate from the ideal periodic metasurface and obtain a quasi-periodic metasurface. In the experiment, we changed the state of the dendritic structures by constantly adjusting the preparation conditions so that the surface with completely disordered dendritic structures was changed to a metasurface with a distinct transmission peak in a selected visible light band. This means that the most probable distribution of the dendritic structures is located in this band, that is, statistically, the distribution of dendritic structures in the metasurface is quasi-periodic.

**Figure 14:**
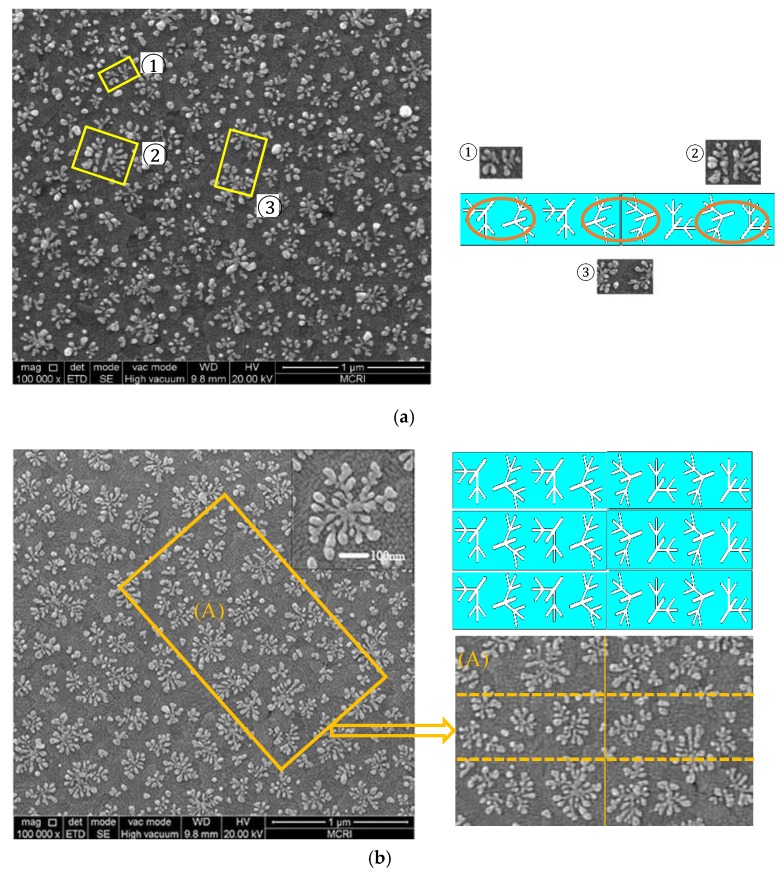
Comparison of the prepared silver dendritic metasurface and designed structures in simulation. Comparing the designed structures with actual dendrites, the dendritic structures in the prepared sample are virtually composed of the designed structures by arranging them up and down and left and right, as shown in (**a**). By observing the SEM image of the sample, it is found that there is a large number of dendritic metasurfaces that resemble the designed metasurface in simulation, as shown in (**b**).

It seems that the dendritic structural material formed by the chemical method was in the random state and not strictly consistent with the dendritic structure in the simulation. However, the distribution of the structure units was quasi-periodic at the macroscopic level, and each dendritic unit grew omnidirectionally. Observing the SEM image of the sample, we found that there are a large number of dendritic structural units similar to those shown in the simulation, as shown in Figure 14. Comparing the structures designed in simulation with actual dendrites in the samples, it can be seen that the rotation of the dendritic structure in the simulation is to make it more identical to the real sample, and the dendritic structures in the prepared sample was virtually composed of the designed structures by arranging them up and down and left and right. Thus, the structural units in the simulation can represent the true condition of the sample, and structural units with radial symmetry need not be designed.

In addition, the area of the incident spot was approximately 4 mm^2^ in the experiment, and the sample area covered by the spot contained approximately 10^7^ dendritic units. Thus, the dendritic structures designed in the simulation must be included. In the simulation, we continued to increase the number of structural units to prove that this anomaly still exists with increasing number of structural units. This step was also performed to illustrate that changing the number of units does not affect the simulation results. We further verified that the phenomenon still persisted when the number of units increased to 10^7^ in experiment. Hence, this theory can be extended to a wider range. Furthermore, metamaterial was a weak interaction system, with very small interaction among structural units. Thus, the interaction between the dendritic units in the dendritic metasurface sample is negligible. A large number of silver dendritic units with structures similar to those in simulation collectively generate responses in the experiment, which is automatic. Therefore, the dendritic units in the metasurface samples were automatically selected to produce anomalous effects during the experimental test. Moreover, the experimental results were statistically significant. The consistency of the simulated and experimental results also coincides with this interpretation. The size of the structural unit in our proposed dendritic metasurface is in nanometer scale and the metasurface can produce a strong electromagnetic response, so it can be used in nanocircuits [38], sensing [39], telecommunications [40] and other fields. Besides, because the dendritic metasurface can produce abnormal optical response, it can also be applied to the field of extraordinary optics such as cloaking [41]. Compared with the technologies mentioned above, the dendritic metasurface responds in the visible light band and can be prepared in large area using a simple, low-cost electrochemical deposition method. And both simulation and experimental results demonstrate that it can achieve plate-focusing in the visible light band.

## 6. Conclusions

We designed a dendritic meta-molecule surface, also called dendritic metasurface, and simulated its optical response behavior in the visible band within the range of 521.7–638.3 nm. This metasurface achieved anomalous reflection and refraction over a wide waveband range. The units of branches were assembled to form a silver dendritic metasurface and realize the focusing effect in the range of 555.6–588.2 nm. The silver dendritic metasurface with different structures and morphologies was prepared through bottom-up electrochemical deposition, which is a simple and cheap method that is applicable in large areas. We also conducted plate focusing in the visible light band. The sample with a transmission peak at 555 nm exhibited a focusing intensity of 11.3% and focal length of approximately 690 µm. The sample with a transmission peak at 580 nm showed the maximum light intensity value in the *z* direction at 560 µm and focus intensity of 6.9%. The sample with a transmission peak of at 650 nm exhibited a focusing intensity of 8.8% and focal length of 595 µm. Although the dendritic structural material formed by the chemical method was in a random state and not strictly consistent with the dendritic structure in the simulation, the number of units in the sample was substantially larger than that used in the simulation. Thus, the optimized dendritic units in the metasurface samples with similar structures to those designed in simulation are to be automatically selected to form the building blocks and then produce anomalous effects. The consistency of the simulation and experimental results also agrees with this interpretation. The metasurface presented in the simulation is only an example, rather than a model, for the prepared sample, although it lead to results in general agreement with the experiment. We will continue to explore the theoretical model of quasi-periodic dendritic metasurface in subsequent research work.
